# Rural community perceptions of antibiotic access and understanding of antimicrobial resistance: qualitative evidence from the Health and Demographic Surveillance System site in Matlab, Bangladesh

**DOI:** 10.1080/16549716.2020.1824383

**Published:** 2020-10-12

**Authors:** Moyukh Chowdhury, Jennifer Stewart Williams, Heiman Wertheim, Wasif Ali Khan, Abdul Matin, John Kinsman

**Affiliations:** a Outcomes Research Department, Reveal AB, Stockholm, Sweden; b Department of Epidemiology and Global Health, Umeå University, Umeå, Sweden; c Research Centre for Generational Health and Ageing, Faculty of Health, University of Newcastle, Callaghan, Australia; d Clinical Research Unit, Oxford University, Hanoi, Vietnam; e Department of Medical Microbiology and Radboud Centre for Infectious Disease, Radboud University Nijmegen Medical Centre, Nijmegen, Netherlands; f International Centre for Diarrhoeal Disease Research, Enteric and Respiratory Infections Infectious Diseases Division, 68, Shaheed Tajuddin Ahmed Sharani, Dhaka, Bangladesh; g Department of Public Health Sciences, Global Health (IHCAR), Karolinska Institutet, Stockholm, Sweden

**Keywords:** Antimicrobial, drug, compliance, qualitative, ABACUS

## Abstract

**Background:**

The use of large quantities of antimicrobial drugs for human health and agriculture is advancing the predominance of drug resistant pathogens in the environment. Antimicrobial resistance is now a major public health threat posing significant challenges for achieving the Sustainable Development Goals. In Bangladesh, where over one third of the population is below the poverty line, the achievement of safe and effective antibiotic medication use for human health is challenging.

**Objective:**

To explore factors and practices around access and use of antibiotics and understanding of antimicrobial resistance in rural communities in Bangladesh from a socio-cultural perspective.

**Methods:**

This qualitative study comprises the second phase of the multi-country ABACUS (Antibiotic Access and Use) project in Matlab, Bangladesh. Information was collected through six focus group discussions and 16 in-depth interviews. Informants were selected from ten villages in four geographic locations using the Health and Demographic Surveillance System database. The Access to Healthcare Framework guided the interpretation and framing of the findings in terms of individuals’ abilities to: perceive, seek, reach, pay and engage with healthcare.

**Results:**

Village pharmacies were the preferred and trusted source of antibiotics for self-treatment. Cultural and religious beliefs informed the use of herbal and other complementary medicines. Advice on antibiotic use was also sourced from trusted friends and family members. Access to government-run facilities required travel on poorly maintained roads. Reports of structural corruption, stock-outs and patient safety risks eroded trust in the public sector. Some expressed a willingness to learn about antibiotic resistance.

**Conclusion:**

Antimicrobial resistance is both a health and development issue. Social and economic contexts shape medicine seeking, use and behaviours. Multi-sectoral action is needed to confront the underlying social, economic, cultural and political drivers that impact on the access and use of antibiotic medicines in Bangladesh.

## Background

Antimicrobial drugs include antibiotics, antifungals, antivirals and antimalarials. Their use in medical and veterinary practice, animal husbandry and agriculture has reduced infectious agents by killing bacteria, fungi, viruses and parasites [1–3]. Exposure to antimicrobials is causing drug resistance. Factors thought to account for this include the suboptimal use of drugs in hospitals, the community, veterinary practice and agriculture. The leakage of compounds into the environment creates pathways for antimicrobial resistance (AMR) although evidence of their relative importance and contribution has not been established [4–6].

**Table 1.** Characteristics of informants in IDIs (n = 16)

	60–80	2	76	Primary	Agriculture, Unemployed
Female	18–60	3	43	Primary to secondary	Homemaker
	60–80	2	61	Primary	Unemployed
	18–30	7	25	Secondary to undergraduate	Homemaker/Care of child under five years

**Table 2.** Characteristics of Informants in FGDs (n = 43)

Sex	Age range(years)	Number of informants	Average Age	Range of Education	Occupation
Male	30–60	7	37	Primary to secondary	Service sector,Agriculture,Business,Teaching
	18–30	6	25	Primary to higher secondary	Student,Business,Service sector
	20–50	8	33	Higher secondary to post graduate	Service sector,Business,Engineer, Unemployed
Female	30–60	8	37	Secondary	Homemaker
	18–30	8	23	Primary to higher secondary	Homemaker
	20–50	6	27	Higher secondary to undergraduate	Homemaker

AMR presents significant challenges for achieving Sustainable Development Goals (SDGs) such as SDG 1 (poverty), SDG 3 (good health and well-being), SDG 6 (clean water and sanitation) and SDG 12 (responsible consumption and production) [7]. AMR is both a health and development issue. Tackling AMR requires an ‘adaptive approach’ that acknowledges how and why antimicrobial use has become entrenched in the way of life in both rich and poor countries [8]. The World Bank estimates that by 2030 up to 24 million people could be forced into extreme poverty due to AMR [9]. Addressing AMR requires acknowledgement of how and why antimicrobials are embedded in societies and economies [8]. The COVID-19 pandemic will further exacerbate this trend which is impacting disproportionately on low- and middle-income countries (LMICs) [10].

The drivers of antimicrobial use in human health are complex and multifaceted [4]. Individual behaviours that promote AMR result from limited knowledge and understanding of potential consequences [11]. A systematic review of studies on AMR showed that addressing the social determinants of poverty is an essential yet neglected step in addressing AMR [12]. In LMICs structural, social, political and economic barriers impede access to prescription medicines, and health system development challenges compromise intervention efforts [8,13,14].

In a qualitative study among human and animal healthcare professionals in Ethiopia, Nigeria, Sierra Leone, India, Vietnam and the Philippines, AMR awareness raising did not reduce prescribing [15]. A study of AMR in rural Thailand concluded that the results of educational programs in high-income countries cannot be generalised to LMICs where impoverished populations endure precarious existence under fragmented under-resourced health and social support systems [16]. In many countries antibiotics are a ‘quick fix’ medicine [8,17]. Balancing access and excess in LMICs poses precarious ethical questions about the roles and responsibilities of users and prescribers [18].

Discussion in the literature has focused on measured ‘use’ and ‘misuse’ of antimicrobials. Researchers in the social sciences remind us of the dangers of injecting subjective bias and unfair judgement into scientific debate [19,20]. Anthropological studies of antibiotic use in Sub-Saharan Africa and South-East Asia used the ‘drug bag’ method to address conceptual and semantic issues [21]. By ‘appropriate’ use we mean that if a medicine is used appropriately (in a clinical sense) it is safe and effective in treating the disease. Conversely, ‘inappropriate’ use occurs when the medicine is not clinically effective in treating the disease. Yet it is important to acknowledge that what is considered ‘appropriate’ from a biomedical perspective, may not be ‘appropriate’ from a socio-cultural perspective [22]. Each country’s ‘risk profile’ for AMR is contextually determined [8].

Bangladesh is a lower-middle-income economy in South-Asia with a population of 163 million https://www.unfpa.org/data/world-population/BD. More than two thirds of the population live in rural areas where social and economic disadvantage and poverty is widespread [2,23–25]. Despite the Government’s National Drug Policy, access to formal healthcare and medical prescribing is limited, and antibiotics are commonly purchased, without prescription, from vendors in pharmacies [23,24,26,27]. A systematic review of AMR studies (2004–2018) in Bangladesh identified a high prevalence of resistance to most antibiotics and major gaps in surveillance [28]. In a country where sixty seven percent of total healthcare expenses are out of pocket and thirty five percent of the population is below the poverty line, achieving safe and appropriate medication use for human health is challenging [29,30].

### Theoretical framework

The Access to Healthcare Framework articulated by Levesque, Harris and Russell [31] is used to guide the interpretation and framing of the findings in this study. The Framework provides a theoretical underpinning because it offers a patient-centred perspective. It goes beyond conceptualising access in a one-dimensional space by presenting and articulating five inter-dependent dimensions and their corresponding abilities at the interface between the population and the health system. The same approach has been undertaken in other similarly designed studies of ABR in low-resource settings [22].

**Figure 1.**
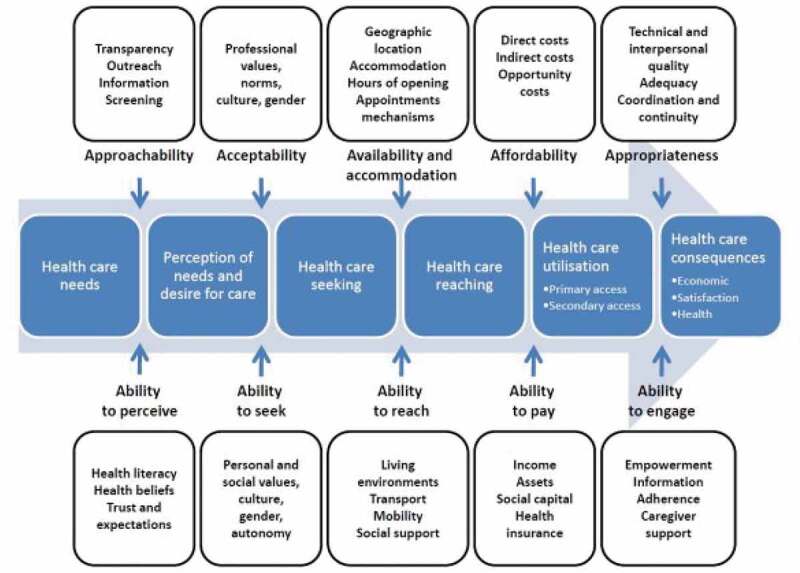
A conceptual framework of access to healthcare developed by Levesque et al. Source: Levesque et al. International journal for equity in health 2013, 12:1

Levesque et. al. [31] propose five dimensions of access: *1) Approachability* (transparency, outreach, information screening); *2) Acceptability* (professional values, norms, culture, gender); *3) Availability* (geographic location, accommodation, hours of opening, appointment mechanisms); *4) Affordability* (direct costs, indirect costs, opportunity costs) *and 5) Appropriateness* (technical and interpersonal quality, adequacy, coordination, continuity). Levesque et. al. [31] proposed five corresponding conceptualisations of the ways in which peoples’ abilities to interact with each of these dimensions are generated: *1) Ability to perceive* (health literacy, health beliefs, trust, expectations); *2) Ability to seek* (personal and social values, culture, gender, autonomy); *3) Ability to reach* (living environments, transport, mobility, social support); *4) Ability to pay* (income, assets, social capital, health insurance) and *5) Ability to engage* (empowerment, information adherence, caregiver support). See Figure 1.

**Figure 2.**
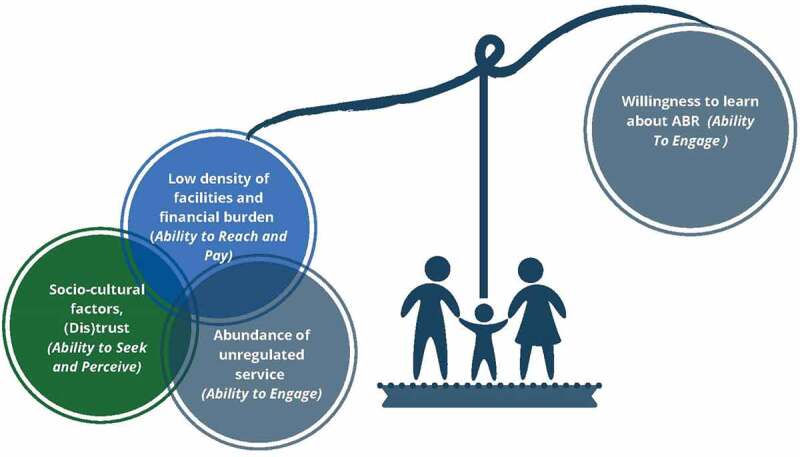
Balancing village perspectives on antibiotic use

### Objective

The objective of this study is to explore factors and practices around access and use of antibiotics and understanding of antimicrobial resistance in rural communities in Bangladesh from a socio-cultural perspective.

## Methods

### Study setting

The study was conducted in the Matlab site of the Health and Demographic Surveillance System (HDSS) in the Chandpur District of Bangladesh. The Matlab HDSS is an economically deprived rural area in southern Bangladesh with an estimated population of 227,935 people in 53,170 households across 142 villages. Further information about Matlab HDSS is published elsewhere [25,32–35] http://ghdx.healthdata.org/record/bangladesh-matlab-health-and-demographic-surveillance-system.

### Study design

This qualitative exploratory cross-sectional study is one component in a larger multi-country research project ABACUS (Antibiotic Access And Use) [36] which assessed and compared community-based antibiotic access and use in Bangladesh, South Africa, Ghana, Vietnam, Thailand and Mozambique. http://www.indepth-network.org/projects/abacus.

ABACUS was conducted in the six countries in two phases (2017–2019). The first phase involved identifying antibiotic resources by mapping suppliers and implementing inventories. In the second phase, factors that affect antibiotic access and use were explored using in-depth interviews (IDIs) and focus group discussions (FGDs) with community members in each of the sites. This study reports the results of 16 IDIs and six FGDs conducted in the Bangladesh Matlab site in the second phase of the ABACUS project. Supplementary file (SF) 1 includes the preparatory in-depth interview guide, SF2 the in-depth questionnaire and SF3 the preparatory focus group guide.

### Sampling and recruitment

Sampling and recruitment followed the ABACUS study protocol under the direction of the third author (HW) [36]. Fourth and fifth authors (WAK and AM respectively) were ABACUS study investigators in the Matlab site. WAK is a senior public health scientist and AM is an anthropologist.

In line with ABACUS protocols [22,37,38] residents were ‘randomly sampled’ from different age and gender categories in ten villages across four different geographic areas identified in the Matlab HDSS database [34,35]. The same method applied in both IDIs and FGDs. Antibiotic suppliers and healthcare workers were not invited to participate. The sixteen IDIs (four men and 12 women) and six FGDs (21 men and 22 women) were conducted between 10 March and 6 April 2017. Four people (two men in the IDIs and one woman and one man in the FGDs) declined to join the study. There were no drop-outs. See Results section for details.

### Data collection

The themes for the qualitative data collection instruments were informed by a review of the literature and developed by JK under the guidance of the ABACUS Principal Investigator (HW). Local support was provided by WK and AM. The interview and focus group questions were developed in English and translated into the Bangla language by WAK and AM. The questions were divided into sections 1) accessing treatment, 2) the supplier/seller of medicines, 3) the medicines. See SFs 1, 2 and 3.

References to specific medicines were made using international brand names which are commonly recognised both in clinical and community settings in Bangladesh. All participants were familiar with the terms, concepts and brand names. The field workers ensured that there were no ambiguities.

The fifth author (AM) conducted all the interviews and discussions with the support of a trained field assistant. Neither were known personally to the informants. All interviews and discussions were tape-recorded. On average, the duration of the IDIs was 45 minutes and the FGDs 90 minutes.

The IDIs and FGDs each had a different purpose. The IDIs were conducted in private homes. Interviewees were encouraged to speak openly about their experiences and give personal opinions. In the FGDs (held in community spaces in villages) there were many opinions being expressed on the same topic. Participants interacted with one another and shared views and insights. Both sets of data were analysed in the same manner.

### Data analysis

Transcripts were recorded in Bangla and translated to English for coding and analysis by the first author (MC) with input from the last author (JK). Transcripts were not shared with the informants.

Data were analysed using a combined thematic approach. By this we mean that the approach was broadly inductive with the concept of access being central and a priori themes derived from the questions used in the FGDs and IDIs. Both sets of data (IDIs and FGDs) were analysed in the same manner. We used Braun and Clarke’s six thematic steps to: become familiar with the data; generate initial codes; search for themes; review potential themes; define themes, and produce a report [39,40]. Codes were generated, assessed and clustered into emergent themes consistent with the Access Framework dimensions [31]. Atlas Ti 7.0 software was used in coding all transcripts.

We were critically aware of the importance of reflexivity during the collection and interpretation of data. The multidisciplinary nature of our team meant that we were cognisant of how different value systems can influence judgement and lead to biased processes and outcomes. The informants were fully informed about the backgrounds of the field workers with whom they had contact.

### Ethical approval

All study informants were fully informed about the purpose of this study, the processes used to collect qualitative information, and the Matlab study’s relationship with the ABACUS project. Informed written consent was a requirement for participation in the IDIs and FGDs. Approval for all ABACUS project sites was granted by the Oxford University Tropical Research Ethics Committee, and the Ethical Review Committee of the International Centre for Diarrhoeal Disease Research, Bangladesh [36].

## Results

### Case study context

Over 40% of people in Matlab live below the poverty line [25]. According to the most recent (2014) Matlab HDSS Household Socio-Economic Census [33] one in six households did not have access to improved water sources, most had mud flooring and tin was the predominant building material. The bicycle was the most common form of transportation. Most men aged 30–54 years were working; the highest proportion of working women (8%) was in the age group 30–44 years. Remittance payments were the main source of income for one third of households. Twenty nine percent of females and 23% of males had not had any formal schooling.

Medical pluralism is an important characteristic of healthcare in Matlab. Residents access formal and informal services for both conventional and alternative medical care and advice. Treatment seeking is influenced by social and cultural beliefs, previous experience and perceptions about the approachability, acceptability, availability, affordability and appropriateness of care [32]. Government-run services charge nominal fees [35]. The most common reported acute illnesses are fever, diarrhoea/dysentery and aches/pains for which self- treatment is common. Matlab’s morbidity and mortality profile is changing from acute, infectious, and parasitic diseases to non-communicable conditions including cardiovascular and cerebrovascular diseases. Longer duration illnesses are managed in government hospitals [32,34].

### Overview of informants

There were 59 informants (25 female and 34 male) from 15 different villages. The age range spanned 18–60 years. The average age of interviewees was 42 years and the average age of focus group participants was 31. Most of the men were employed and most of the women were engaged in full time home duties. Islam was the dominant religious faith. None of the informants held health insurance (see Tables 1 and 2).

### Dimensions of access

Data are presented under the five thematic dimensions proposed by the Access Framework developed by Levesque et. al. [31]. As there were no substantial differences in the results of the IDIs and FGDs, the findings are grouped togther.

#### Approachability/ability to perceive

‘Approachability’ relates to how individuals perceive the existence of services they need. Village pharmacies were directly approachable for primary treatment. Young women sought treatment based on what they believed had worked well in the past.

We usually go to the nearby pharmacies for primary treatment. If anything is serious then we travel to the Upazilla (approximately nine kilometres). The treatment at the pharmacy works fine, that’s why I barely go to the hospital. Moreover, I get medicines in credit sometimes’. (IDI – female, age 23, secondary education, homemaker).

A mother commented that she used medications she could trust to provide a ‘quick-fix’.

If there is any trouble I take an extra dose of antibiotic. If I am having stomach-ache today, I will need a pill. So if I take one dose extra, then there will be no pain (IDI – mother of child under five years, age 30, secondary education, homemaker).

Men referred to serious issues of structural violence in public sector facilities. In contrast, village pharmacies offered a safer trusted alternative.

‘We go to private facilities because of the poor service in the public ones. Even though the treatment is free in public facilities, they do not treat well without bribe or connection. Moreover, often we do not even get free medicines. They just sell them outside. We are treated with negligence. If we go to the public healthcare centres, we might end up dying without treatment.’ (FGD – male, age 50, secondary education, agriculture).

Women mentioned limited medicine supply as a further impediment.

‘We do not have any government health facility in our area. There is one in our neighbouring village but the government ones do not always have a sufficient supply of medicines’. (FGD – female, age 22, primary education, homemaker).

Informants self-medicated with antibiotics for: wound healing, headache, lethargy, blood pressure control, urinary tract infection and stomach ache. Commonly recognised brand names were: *Fimoxyl, Zthrin, Zmax, Cephrad and Cef-3*. Health literacy and trust was built on familiar trusted brand names.

#### Acceptability/ability to seek

There were several choices to make regarding where to seek acceptable healthcare. Non-medical spiritual practices are important healing methods in the local belief system. These methods included *‘Jharfuk’* (sorcery involving blowing holy verses), *‘Kabiraji’* (treatment with herbal extracts), *‘Tabij’* (an amulet containing verses from holy books believed to be protective and curative against diseases) and *‘Pani Pora*’ (water with spell from the religious pastor which is believed to be curative).

‘Kabirajs do not give medicines, they give “Tabij” instead, and people recover there. As we believe in religion, we believe that Allah can cure us without medicines if he wants, he can save us without treatment’. (FGD – male, age 30, higher secondary education, service sector employment).

Younger people were aware of religious discrimination in government healthcare. This influenced their ability to seek healthcare.

‘The hospital is in MND which is a Hindu area. So people from our area do not really prefer going there.’ (FGD – male, 30, higher secondary education, business sector employment).

Medicines were obtained (free of charge) from friends, relatives and neighbours. Having personal contacts with hospital or pharmacy experience enhanced self-efficacy.

‘I do not need to visit doctors mostly as my neighbour works in the hospital and gets free medicines from there. I take antibiotics from her, even for my family members. She tells me how to take it’. (IDI – mother of child under five years, age 22, undergraduate education, homemaker).

A young wife had full confidence in her husband’s advice because of his village pharmacy experience.

‘In my family, my husband suggests which antibiotic is to be taken as he has a good knowledge about medicines. He used to work in a pharmacy and is involved in a pharmacy business now’. (IDI – female, age 30, higher secondary, homemaker).

#### Availability/ability to reach

The ability to reach healthcare is related to the ability to seek healthcare. Men aired complaints regarding the distance to hospitals. Village based services were available and easy to reach.

‘There is only a community clinic in our village. The nearest government hospital is there in ML (another village 8 kilometers away from the village) and then Matlab (sub-district centre 15 kilometres from the village)’. (FGD – male, age 38, primary education, agriculture).

Public infrastructure was inadequate on multiple fronts – roads, facilities and the medical workforce.

‘We do not have sufficient physicians in our area. For primary care, we need to go to DK (around 10 kilometres away from his village). They are really far, moreover, look at the condition of the roads. We need more doctors and better facilities for treatment for our people’. (FGD – male, age 37, primary education, service sector employment).

At the very least it was important to ensure that village residents could reach medical facilities to obtain treatment when needed. Another male focus group informant made the following comment.

‘Government health facilities are far from here, also look at the condition of the roads. This is hard to travel. We need doctors and better treatment for our people’. (FGD – male, age 31, higher secondary education, business sector employment).

#### Affordability/ability to pay

Even though most medicines are free of charge in government-run facilities, informants preferred to use trusted nearby village pharmacies where they could purchase medicines quickly over the counter. Yet personal economic circumstances influenced ability to pay.

‘Antibiotic medicines cost around 70 takas ($US 0.8) per day and around 150 takas ($US 1.6) for two days. I do not have any other financial support, so how can I afford this? Think about my situation. If a man cannot afford to buy milk and eggs, how can he afford to buy full doses of antibiotics?’ (FGD – male, age 50, primary education, agriculture).

Men shared personal accounts of their healthcare expenditure and monthly earnings in the focus group.

You have to accept the reality. If you earn 5000 BDT/month ($US 62) you cannot afford to pay healthcare costs at all if the problem is more than just a cold or a fever. Many people in the village struggle with expenses for treatments. Some people borrow money and others end up selling all their lands and properties to afford to pay treatment costs’. (FGD – male, age 50, higher secondary education, unemployed)

#### Appropriateness/ability to engage

Pharmacy healthcare was appropriate. Village residents were comfortable engaging with local salespersons in pharmacies.

‘Frankly speaking, our healthcare system is totally pharmacy-based.’ (FGD – male, age 28, higher secondary education, service sector employment).

Pharmacy salespersons hold a level of authority and accountability with regard to medication advice. They are trusted and respected by both men and women. Salespersons typically cut slits in the boxes to help those unable to read. Three cuts, for example, can mean one pill three times a day.

‘They (pharmacy salespersons) tell us how to take the medicines and we depend on this advice. If they give something wrong, they will be in trouble. They might not be MBBS doctors but they are from our area, so we trust their management.’ (IDI – mother of child under five years, age 30, secondary education, homemaker).

Antibiotics were perceived as being useful for treating infections. But there was also a belief that antibiotics could be used to treat non-infectious conditions such as hypertension and lethargy. Some male and female focus group participants volunteered views on resistance.

‘Antibiotic resistance? I heard that antibiotics will be banned or something, on the TV news. It said around hundred companies were banned or something. I cannot remember correctly.’ (FGD – male, age 42, postgraduate education, business sector employment).

There was curiosity about correct dosages.

‘Yes, we have that habit. If we take antibiotics for two days and get well, we stop taking it. This causes the problem and the disease recurs. Who knows? Frequent use of antibiotics might turn out to be another disease.’ (FGD – female, age 27, higher secondary education, homemaker).

Awareness raising activities were discussed. Examples include audio-bulletins in villages, neighbourhood discussions, door-to-door promotions by community health workers, counselling by physicians and advertising in the media (television, radio and newspapers). Treatment seeking was women’s responsibility and their role was important in raising awareness about antibiotics and AMR.

‘I think the females of the community should be focused to educate about the issue as the males are usually busy. The female members of the family can teach them later’. (IDI – female, age 62, primary education, unemployed).

## Discussion

The findings of this qualitative study highlight multiple factors and practices around the access and use of antibiotics and the understanding of ABR in Matlab, Bangladesh. Healthcare seeking is embedded in social, economic, political and institutional structures and belief systems. Individuals in resource-poor settings have limited capacity to make the same evidence-based choices that are available in more advantaged populations [13,17,41]. ‘Structural violence’ in government facilities inhibits access to the public sector for antibiotic medicines [42]. Local retail pharmacies and clinics are the accepted primary healthcare choice. Advice regarding antibiotic medicines is based on common practice and reinforced by trust built on personal communication and local knowledge [32,35,43].

According to the Access to Healthcare Framework [31] impediments to accessing prescription antibiotic medicines result from: the low density of facilities and the financial burden of purchasing antibiotics (*availability/ability to reach* and *affordability/ability to pay*); socio-cultural factors and trust (*acceptability/ability to seek* and *approachability/ability to perceive)* and the abundance of unregulated services (*appropriateness/ability to engage*). Enablers capture the willingness to learn (*appropriateness/ability to engage*). See Figure 2.

Most were unaware of the term ‘antibiotic resistance’ or ‘ABR’ although some understood the link between ‘inappropriate’ use and ‘effectiveness’. Many reported a *willingness to engage* in learning about AMR. *Health literacy* was built from past experience. ‘Trusted’ brands that had provided a ‘quick-fix’ in the past were preferred.

Over sixty percent of patients’ total healthcare expenditure is for pharmaceuticals, and over sixty percent of this is borne out of pocket [29]. The average gross income per capita in Bangladesh is only $US 3 per day http://povertydata.worldbank.org/poverty/home/, The price per capsule of the three most popular antibiotics Zithrin, (Azithromycin), Zmax (Azithromycin) and Cef-3 (Cefixime) is about $US 0.40. A standard course of any of these medicines costs between two and six $US. When medication costs impact on ability to pay there is an incentive to under-dose if this saves money.

As is the case in most LMICs, the informal sector in Bangladesh provides the bulk of healthcare for the poor [44]. A systematic review of the role of informal providers in developing counties cited convenience, affordability and social and cultural norms as the main reasons for their popularity [45]. References to the informal sector, whether in housing, the labour market or healthcare are often made in a normative context, imposing unfair judgment and discrimination on disadvantaged and marginalised populations for whom these services offer the most affordable and suitable option [46]. In this study, informants reported self-medicating with antibiotics because they believed, in good faith, that these medicines would alleviate symptoms and lead to recovery [17]. Our findings illustrate how the *acceptability* of village pharmacies as providers of antibiotic medicines is embedded within the social and cultural fabric. People valued the *accessibility* of local stores and were reassured by the *approachability* and familiarity of trusted pharmacy salespersons. Care seeking behaviours were motivated by good intent to remedy immediate health concerns in an expedient affordable manner [11].

Family budgets were constrained. Healthcare was covered after providing food and shelter. Even though antibiotics were nominally ‘free of charge’ when purchased from government facilities, there were service charges to consider. Poorly maintained roads made people reluctant to travel to hospitals located several kilometres away when they could purchase medications and obtain healthcare advice locally.

Trust plays an important role in healthcare *approachability* [31]. Trust in government run services was eroded by perceptions of bribery, corruption, negligence and the scarcity of medicine supplies. Some perceived patient safety to be at risk. Informants therefore trusted pharmacy sales persons more than medical practitioners. It is essential for policy-makers to understand the underlying reasons for this trust imbalance. Health sector reforms need to accommodate the roles played by both providers and seekers within a fractured pluralistic healthcare system [32].

The availability and affordability of quality-assured medicines is an essential requirement for the delivery of primary healthcare [47]. The commonly accepted purchasing of medicines from village pharmacies must be understood in context. The vulnerability of individuals due to circumstances such as hardship, poverty, and stress impacts on access to healthcare and medicines in ways that are often not well understood by policy-makers and regulators operating from positions of relative advantage and prosperity.

### Strengths and limitations

The study was conducted in Matlab, an HDSS site since 1963 and home to numerous significant public health interventions in Bangladesh [34,35]. The ABACUS project provides an empirical basis for understanding antibiotic use and informing context specific interventions in six LMICs [22,37,38,43,48]. The research infrastructure is well established, and we are confident that all ethical and scientific protocols were followed correctly over the course of this study. The data collection methods were standardised across the six-country study sites in the ABACUS project [36]. Although there were opportunities for some probing questions we followed the pre-defined topics. See SF1, SF2 and SF3. This study in Matlab provides rich contextual insights into how and why people’s medicine behaviours are as they are.

However we do acknowledge that there may be a degree of fatigue due to the area being well-researched by the DHSS over several decades. Moreover, residents across Matlab may be more astute about the issues discussed here than people in other rural parts of Bangladesh. The findings are not intended to be representative, generalizable or comprehensive.

### The way forward

Antibiotic use in Matlab villages reflects a fractured public healthcare system that remains out of touch with the issues that determine health seeking behaviours and oblivious to the erosion of trust in public infrastructure and services. Future research in Matlab might include ethnographic methods such as participant observation to explore further lines of enquiry regarding the way underlying social, economic and political determinants underpin behaviours [17].

The focus on individual behaviour change needs to be complemented by attention to the dynamic complex processes responsible for knowledge acquisition. We need to understand more about the ways in which social, cultural and economic factors impact on the knowledge, attitudes, beliefs and behaviours of patients, health professionals and the broader public in regard to the use of antimicrobials in human health and agriculture [49,50].

Cross discipline research, using mixed methods, is needed to focus on the dynamic interplay between contextual constraints, communication and behaviour change among the various agents involved in care seeking. Informal providers are a neglected yet important group in AMR research. There is a need for research to unpack the issues from the perspective of informal providers to help inform strategies to build community trust in government-run health services [45].

Awareness raising campaigns have had limited success in LMICs because they have tended to focus on, and also judge, knowledge deficits [16,22]. Yet mainstream public health education messages are not appropriate for those experiencing hardship and abject poverty on a daily basis [11]. Educational campaigns need to be complemented by upstream drivers of AMR such as poverty and unemployment and structural violence [12,14].

At the national level, the implementation and enforcement of the Bangladesh Government’s National Drug Policy must be strengthened [23,24,26,27]. The World Health Organization acknowledges the need to increase awareness and knowledge about antibiotic medicines and ABR more broadly in society. Specifically this means developing surveillance and research, enhancing infection control, optimizing the use of antimicrobials in human and animal health, and building sustainable investment in new medicines, diagnostic tools, and vaccines [51].

## Conclusion

Progress will only be achieved by understanding human behaviours and actions in relation to norms, assumptions, beliefs and attitudes at the intersection between social and economic circumstances and political power structures. Interventions aimed at mitigating AMR must address the entrenched social, economic, political and cultural conditions in which people live, work and seek care [12,16]. Sustainable solutions will require multi-sector national action plans with clear targets and lines of accountability to ensure that political will translates to effective action [8]. Accountability for AMR lies with governments and global authorities both within and beyond the health sector [18,52].

## Paper context

Antimicrobial drugs (antifungals, antivirals and antimalarials) are public goods. Inappropriate use promotes the spread of resistant pathogens and reduces treatment efficacy, thereby impacting on public health. In Bangladesh, over sixty percent of patients’ total healthcare expenditure is for pharmaceuticals, over sixty percent of which is borne through out-of-pocket purchasing of antibiotics from unregulated vendors without medical prescription or advice. This study found that people in rural communities in Matlab were either not informed or knew little about appropriate access and use of antibiotic medicines. The principle means through which knowledge was available was village pharmacies.
